# Advancements in antiviral approaches against foot-and-mouth disease virus: a comprehensive review

**DOI:** 10.3389/fvets.2025.1574193

**Published:** 2025-07-16

**Authors:** Mahmoud Mohamadin, Rashid Manzoor, Ahmed Elolimy, Mohamed Abdelmegeid, Samah Mosad, Sahar Abd El Rahman

**Affiliations:** ^1^Department of Virology, Faculty of Veterinary Medicine, Mansoura University, Mansoura, Egypt; ^2^College of Veterinary Medicine, University of Al Dhaid, Sharjah, United Arab Emirates; ^3^Veterinary Science Program, Faculty of Health Sciences, Higher Colleges of Technology, Sharjah, United Arab Emirates; ^4^Department of Integrative Agriculture, College of Agriculture and Veterinary Medicine, United Arab Emirates University, Al Ain, United Arab Emirates; ^5^Department of Animal Medicine, College of Veterinary Medicine, Kafrelsheikh University, Kafr El Sheikh, Egypt

**Keywords:** foot-and-mouth disease, antiviral approaches, vaccination advancements, novel therapeutics, future challenges

## Abstract

Foot-and-mouth disease (FMD) is a highly contagious viral disease that poses a significant threat to the global livestock industry. Despite extensive vaccination efforts, outbreaks continue to occur frequently, highlighting the need for effective therapeutic interventions. This review comprehensively examines the recent advances in antiviral therapies targeting the foot-and-mouth disease virus (FMDV), alongside an overview of recent developments in FMD vaccines. We extensively reviewed the published literature on various antiviral agents targeting FMDV, including small-molecule inhibitors, biologics, RNA-based therapeutics, gene delivery systems, and innovative approaches such as virus protease inhibitors and nanomaterials. Among the notable findings are the promising results of monoclonal antibodies, porcine interferon-*α* fusion proteins, and RNA interference (RNAi)-based therapeutics. This review also discusses the antiviral properties of naturally derived substances, such as quercetin and homoharringtonine, and their mechanisms of action against FMDV. The efficacy of these antiviral agents in inhibiting FMDV replication has been demonstrated by both *in vitro* and *in vivo* studies, underscoring their potential as adjunctive tools in FMD control. Despite these advancements, challenges persist, including the emergence of drug-resistant strains, limited *in vivo* efficacy, and lack of approved antivirals for FMD. This review critically analyzes the advancements in both vaccines and antiviral compounds against FMDV. Continued research is essential to optimize antiviral candidates, address emerging challenges, and improve overall response efforts to FMDV infections.

## 1 Introduction

Foot-and-mouth disease virus (FMDV) belongs to the Picornaviridae family and is characterized by its positive-sense RNA genome and high genetic variability (1). FMDV isolates are classified into seven distinct serotypes: O, A, C, Asia 1, and South African Territories (SAT 1, 2, and 3). Each serotype exhibits unique serological characteristics and contains various genetically and geographically diverse subtypes, often referred to as topotypes (2). FMD outbreaks lead to considerable economic losses due to trade restrictions, livestock mortality, and reduced productivity (3).

The control of FMDV remains challenging due to its high genetic variability, which results in the emergence of antigenically distinct viral strains that can evade existing immune responses generated by current vaccines (4). The high mutation rate of the RNA genome contributes to this genetic variability, complicating the development of effective vaccines and antivirals. The rapid evolution of FMDV strains and the emergence of vaccine escape variants underscore the need for complementary control measures, including the use of antiviral drugs, despite the widespread implementation of vaccination campaigns (5). Thus, it is essential to investigate and develop antivirals that target various stages of the FMDV life cycle, offering an additional layer of defense alongside vaccination efforts.

In this review, we examine the advancements in antiviral approaches and vaccines against FMDV, emphasizing the most promising compounds and their mechanisms of action. We also examine the potential of combining antiviral agents with vaccination to enhance protection against this highly contagious viral disease.

## 2 FMDV structure and replication

**Figure 1:**
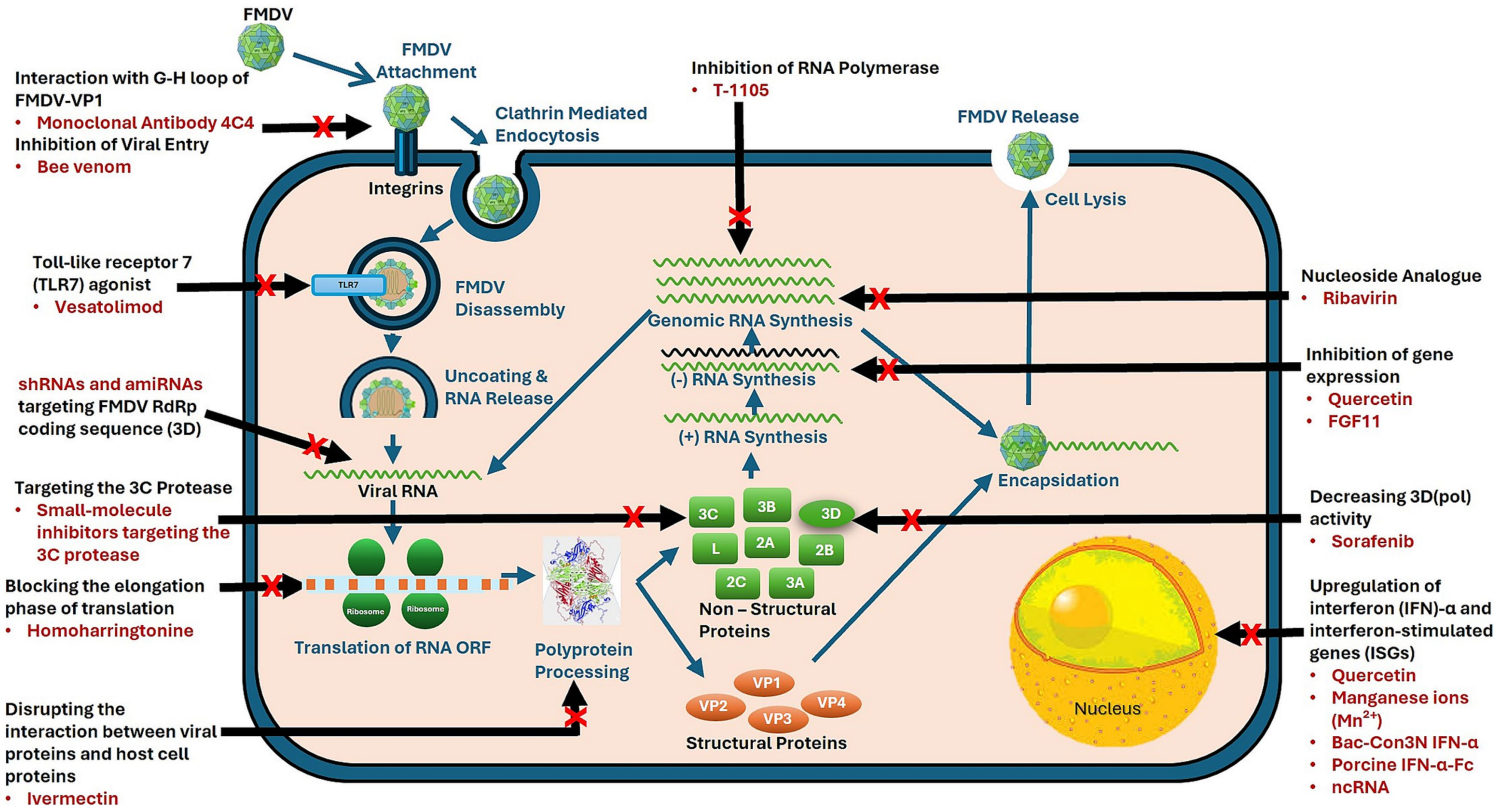
Mechanisms of action of antivirals targeting foot-and-mouth disease virus (FMDV). FMDV lifecycle highlights stages targeted by antiviral agents. Red crosses indicate specific intervention points by antiviral agents, targeting processes such as viral entry, genome replication, and virion assembly to inhibit viral propagation.

FMDV is a nonenveloped virus with a single-stranded RNA genome of approximately 8,400 nucleotides. The genome contains a single open reading frame that encodes a polyprotein. This polyprotein is cleaved by the viral proteases leader protease (Lpro) and 3C protease (3Cpro) to generate several polypeptides: P1 (comprising VP1–VP4), P2 (including 2A, 2B, and 2C), and P3 (comprising 3A, 3B, 3Cpro, and 3Dpol). These polypeptides are processed to yield four mature structural proteins (VP4, VP2, VP3, and VP1) and eight nonstructural proteins (Lpro, 2A, 2B, 2C, 3A, 3B, 3Cpro, and 3Dpol) (Figure 1), all of which are essential for host cell infection and immune evasion (6, 7).

Several key FMDV proteins play crucial roles in viral replication. The structural proteins VP1, VP2, VP3, and VP4 assemble to form the viral capsid, which mediates host cell attachment and invasion. The nonstructural protein 3Cpro processes the viral polyprotein and enables viral replication, making it an important target for antiviral drug development. Lpro interferes with the innate immune response by cleaving specific host proteins. RNA-dependent RNA polymerase (3Dpol) mediates viral RNA genome replication, making it a critical target for antiviral drugs (8, 9).

The FMDV replication cycle begins with viral attachment to specific host cell surface receptors, followed by endocytosis and uncoating of the viral RNA genome. FMDV primarily uses integrins, such as αvβ3 and αvβ6, as receptors for host cell entry. These receptors recognize the RGD (Arg-Gly-Asp) sequence in the VP1 protein of FMDV, facilitating viral attachment and entry, particularly in epithelial tissues. Some FMDV strains also bind to heparan sulfate, expanding the virus host range (10). These interactions are key to viral infectivity and are essential for developing antiviral therapies.

## 3 Economic impact of FMD on livestock industries

FMD is a highly contagious viral disease that affects cloven-hoofed animals, including cattle, pigs, sheep, and goats (11). It has a considerable economic impact on livestock industries, leading to substantial financial losses due to decreased productivity, trade restrictions, and high costs associated with disease management and eradication (12).

The annual economic impact of FMD, including visible production losses and vaccination costs in endemic regions, ranges from US$6.5 to US$21 billion. Outbreaks in FMD-free countries and zones incur additional annual losses exceeding US$1.5 billion (3).

In the context of agroterrorism, FMD is considered a high-risk agent due to its ease of transmission and potential to cause widespread economic disruption. The deliberate introduction of FMD into a country with a susceptible livestock population may devastate the agricultural sector, leading to food shortages, inflation, and economic instability. To prevent such circumstances, robust biosecurity measures, early detection systems, and effective response strategies are required (13).

## 4 Global strategy and implementation of FMD control

Effective control of FMD involves strict biosecurity measures, vaccination programs, and surveillance. Biosecurity measures, which include controlling animal movement and enforcing quarantine protocols, are crucial in preventing the introduction and spread of FMDV within and between farms (3). Regular and widespread vaccination campaigns are necessary to maintain herd immunity and reduce the risk of outbreaks (14). Additionally, continuous surveillance and early detection systems enable rapid response to outbreaks, helping to contain the disease before it spreads widely (15).

The Global Foot-and-Mouth Disease (FMD) Control Strategy, established in 2012 by the World Organization for Animal Health (WOAH, formerly OIE) and the Food and Agriculture Organization of the United Nations (FAO) as part of the Global Framework for the Progressive Control of Transboundary Animal Diseases (GF-TADs), aims to alleviating FMD’s impact in affected regions while supporting nations in either maintaining or achieving WOAH-recognized FMD-free status (16). The cornerstone of this global strategy is the Progressive Control Pathway for FMD (PCP-FMD), developed collaboratively by the FAO and the European Commission for the Control of Foot-and-Mouth Disease (EuFMD) and subsequently endorsed by WOAH (17). This framework implements a systematic, evidence-based approach to risk management, designed to gradually reduce disease impact and viral presence in endemic areas. The PCP-FMD framework acknowledges and accommodates the diverse stages of progress across different nations, ranging from those with established FMD-free status to those in the initial phases of implementing control measures (18).

Vaccination serves as a fundamental component within the PCP-FMD framework, playing a crucial role in reducing disease occurrence and interrupting viral transmission patterns. The success of vaccination programs depends significantly on ensuring that vaccines are well-matched to circulating viral strains (19). The vaccination strategy encompasses multiple objectives that vary according to the specific stage of disease control, including (i) reducing the clinical incidence of FMD, (ii) eliminating the circulation of FMD virus (FMDV), (iii) maintaining FMD-free status, or (iv) regaining freedom from FMD after an outbreak. A critical aspect of the PCP-FMD framework, particularly in stages 2 and 3, is the implementation of comprehensive post-vaccination monitoring (PVM) and population immunity surveillance. These monitoring systems are crucial for countries adopting vaccine-based strategies to control FMD effectively (20).

## 5 Advancements in vaccine development against FMDV

**Figure 2:**
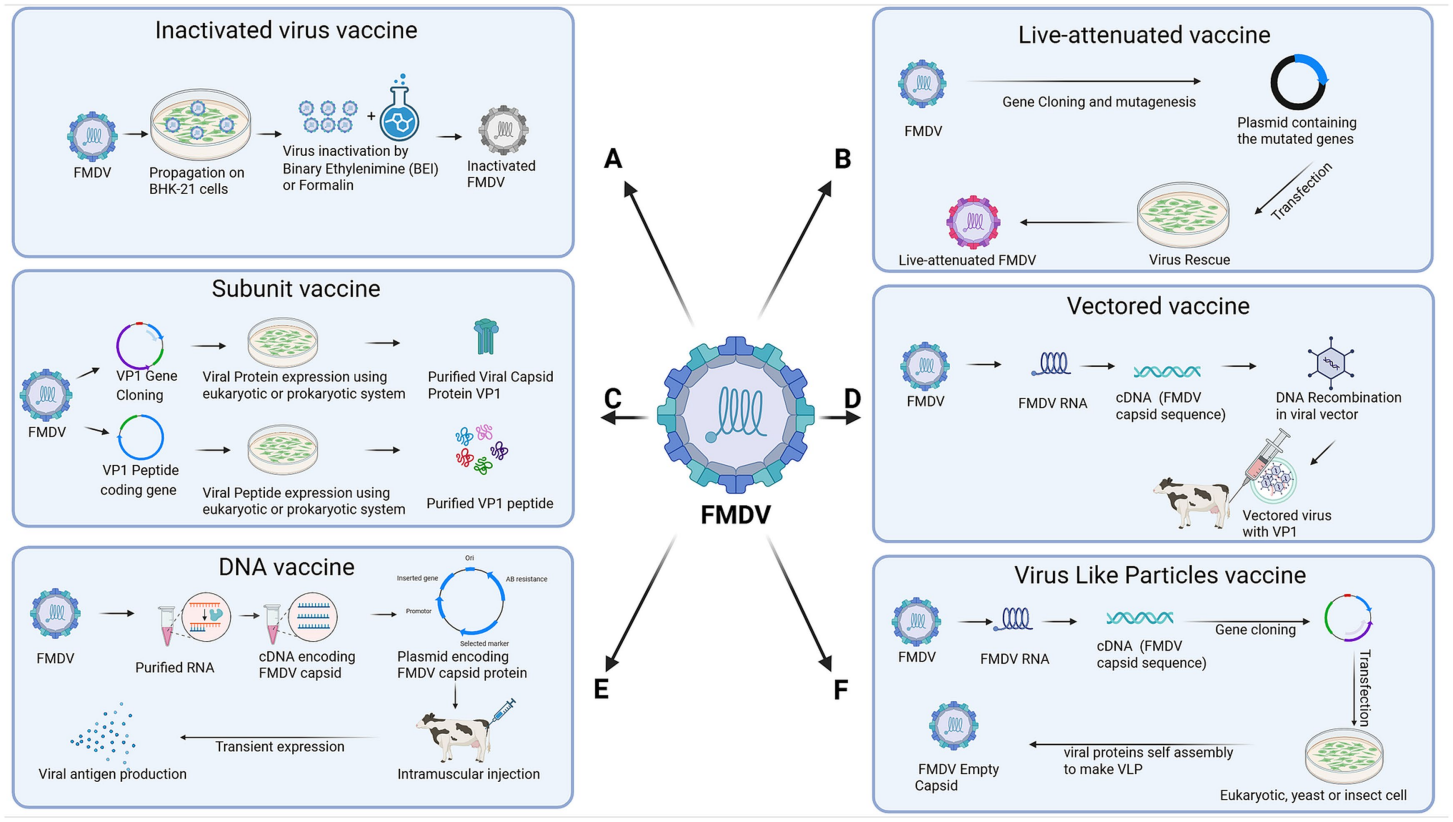
Advancements in vaccine development against foot-and-mouth disease virus (FMDV). This figure illustrates the design of six main types of FMDV vaccines. **(A)** Inactivated virus vaccine. **(B)** Live-attenuated vaccine. **(C)** Subunit vaccine. **(D)** Vectored vaccine. **(E)** DNA vaccine. **(F)** Virus-like particles (VLP) vaccine. The figure was created with Biorender.com.

Inactivated vaccines, which contain killed virus particles, are widely used because of their safety and ability to induce protective immunity. These vaccines stimulate the production of neutralizing antibodies, which are crucial for preventing FMDV infection and spread (21). Despite their effectiveness, several factors limit the utility of FMDV vaccines. One significant challenge is strain-specific protection, as the high genetic variability of FMDV leads to the emergence of new strains that may not be adequately protected by current vaccines, necessitating frequent updates to vaccine formulations. The immunity provided by inactivated vaccines is of limited duration, requiring regular booster doses to ensure continued protection. Furthermore, the necessity for a consistent cold chain during storage and transportation presents logistical difficulties, especially in areas with limited infrastructure (15). Recent advancements in vaccine technology aim to address these limitations. Novel vaccine platforms, including virus-like particles (VLPs), peptide-based vaccines, and recombinant vaccines, are being explored for their potential to provide broader and longer-lasting immunity. Adjuvants are also being explored to enhance vaccine efficacy and induce stronger immune responses (22). In the following sections, we shall discuss various types of vaccines that have been tested for their potential to prevent/control FMD. Figure 2 provides an overview of the key advancements in FMDV vaccine development.

### 5.1 Inactivated (killed) vaccines

Inactivated or killed vaccines, produced as monovalent, bivalent or multivalent, have long been used for the control of FMD (22). Recent advancements in inactivation methods and adjuvant formulations have greatly enhanced the efficacy and safety of inactivated vaccines. Binary ethyleneimine–mediated inactivation ensures reliable virus inactivation while preserving antigenic integrity, thereby increasing vaccine immunogenicity (23). Conventionally, killed vaccines are concentrated to the equivalent of three times the 50% protective dose (PD50%). Killed vaccines are concentrated to the equivalent of six times PD50 for emergency use in FMD vaccine free countries (24). Novel adjuvants for FMD vaccines have been developed to boost the immune response, conferring extended protection with fewer doses. Successful adjuvants include mineral oils such as montanide ISA-206 and ISA-201, aluminum hydroxide, saponins like Quil-A, and Toll-like receptor ligands targeting pattern recognition receptors (23). Furthermore, improvements in cold chain logistics and vaccine stability have made inactivated vaccines more accessible in regions with limited infrastructure, enhancing their global application (22). Advances in multivalent vaccine formulations now enable broader protection against multiple FMDV serotypes, addressing one of the major challenges in FMD vaccination (25).

### 5.2 Live attenuated vaccines

Recent advancements in genetic engineering and viral attenuation techniques have reignited interest in the use of live attenuated vaccines for FMD despite previous restrictions due to safety concerns. Modern live attenuated vaccines are developed using precise genetic modifications that reduce the risk of reversion to virulence (26). Advancements in understanding the role of various viral proteins in pathogenesis have played a crucial role in developing these attenuated vaccines. For example, the deletion of the gene encoding viral leader protease (Lpro) that blocks innate immune response via blocking beta interferon induction made the virus avirulent for pigs and cattle (27). So far, various attenuated vaccines have been developed and tested, including generation of a leaderless virus (LLV), deletion of Lpro, excision of the conserved SAP domain from Lpro, Chimera of FMDV and bovine rhinitis B virus (BRVB) and codon pair optimization (28–30, 31, 32). Additional improvements in vector design and delivery methods have strengthened the safety profile of these vaccines. These innovations have the potential to produce highly immunogenic vaccines that can induce robust and long-lasting immunity with fewer doses. However, ongoing research is necessary to fully evaluate their safety and efficacy in large-scale applications (33).

### 5.3 Subunit vaccines

The identification of key immunogenic proteins of FMDV has driven significant progress in the development of protein-based and peptide-based subunit vaccines through the use of recombinant DNA technology (26). Once it was confirmed during the 70s that VP1 had prominent exposure on virus surface against which neutralizing antibodies are produced, attempts were made to develop protein-based vaccines as an alternative to inactivated vaccines (34, 35). Subsequent studies showed that VP1-based vaccines, prepared from split virus particles or recombinant DNA technology, conferred protection against FMDV challenge in swine and cattle (36).

With the increase in understanding of the immunogenic epitopes in VP1, peptide-based vaccines emerged. Initially, peptide-based vaccines comprised of C terminal half or G-H loop of VP1 (37). The main advantage of peptide-based vaccines is in their ability to stimulate a targeted immune response while avoiding the use of live virus, thus enhancing safety. However, their main limitations were poor immunogenicity/protection *in vivo*. Therefore, different methods have been tested to enhance the immunogenicity of peptide-based vaccines. Some of these included optimizations of B and T cells sites in peptide, combining vaccine with adjuvants, a multiepitope recombinant chimeric protein consisting of different FMDV variants and one T-cell epitope or the use of dendrimer peptides as vaccines (38–40). Despite these challenges, peptide-based vaccines have shown promise in preclinical and clinical studies. A synthetic peptide-based vaccine (UBITh vaccine) has been developed by United Bomedical Inc. and licensed for use in Taiwan and mainland China for the prevention of FMD in pigs. Further research is needed to optimize their formulation and assess their long-term efficacy in diverse animal populations (41).

### 5.4 Vectored vaccines

Vectored vaccines deliver the sequence of interest, i.e., a viral protein, peptide or an epitope to stimulate ideally both humoral and cell mediated immune responses. A number of viruses, including vaccina virus, fowl pox virus, pseudorabies virus, alphaviruses, replication- defective human adenovirus virus, simian adenovirus, and Semliki Forest virus have been used as vectors showing various pros and cons. A recombinant Sendai virus expressing FMDV P protein induced protective immunity in vaccinated mice (42). Vaccination with recombinant infectious bovine rhinotracheitis virus (IBRV) expressing FMDV-VP1 has been shown to induce protective titers in calves (43). Bovine enterovirus expressing an FMDV-VP1 epitope has also been reported, though not tested by a challenge protection study (44).

A limiting factor in using host-specific virus-based vectors is the presence of pre-existing antibodies that limit the virus replication, thus resulting in low antibody titers. Another way to avoid neutralization by pre-existing antibodies is the use of non-host specific virus-vectored vaccines. Bamboo Mosaic virus expressing VP1 antigenic epitope provided protection in swine against challenge (45). Canine adenovirus expressing FMDV-VP1 protein induced humoral response in pigs (46). A number of studies have shown the usefulness of human adenovirus-based vaccines against FMD, such as the replication-deficient human adenovirus vectored FMD (AdtA24) vaccine (47), bivalent Ad5A24 + O1 vaccine (48), adenovirus 5 (Ad5) vectored FMDV serotype O1-Manisa subunit vaccine (Ad5-O1Man) (49) etc.

Advances in genetic engineering have enabled the incorporation of multiple FMDV antigens into a single vector. A recombinant Ad5-vectored vaccine expressing VP1 of O, A and Asia-1 serotypes was tested. The study showed that this trivalent vaccine showed poor protective efficacy in cattle compared to Ad5-vectored monovalent vaccines (50). The advantage of using human adenovirus-based vaccines is DIVA capability, induction of both humoral and cellular responses and easy mass-production. Furthermore, improvements in vector delivery methods, including intranasal and intradermal administration, have enhanced the immune response by targeting mucosal immunity, which is crucial for protecting against FMDV (51).

### 5.5 DNA vaccines

DNA vaccines represent a cutting-edge approach for FMD prevention, with significant advancements in vaccine design and delivery. DNA vaccines are usually plasmid-based vaccines containing the gene of interest and promoter for gene expression and immunity induction. DNS-based vaccines have been tested against many bacterial, viral, and parasitic disease models and tumors. Several strategies in developing DNA vaccines against FMD have been tested, including DNA vaccines encoding VP1 and 3D RNA polymerase (52), a DNA vaccine producing antisense RNA binding with 5′ UTR and expressing the VP1 protein (53), a DNA vaccine expressing B and T cells epitopes directed to antigen-presenting cells or swine MHC class-II antigen (54) and co-expression of anti-apoptotic protein Bcl-Xl with FMDV B and T cells epitopes (55). The main challenges with DNA vaccines are the large amount of DNA needed and repetitive inoculations to achieve protective titers. Techniques such as electroporation, which uses electrical pulses to increase cell membrane permeability, have greatly enhanced the delivery of plasmid DNA into host tissues, eliciting stronger immune responses (56).

### 5.6 Virus-like particle (VLP) vaccines

Virus-like particle vaccines have become a promising approach for FMD prevention because they closely mimic viral structures but lack any viral genetic material. Recent advancements in VLP production technology, including recombinant expression systems in yeast, insect, and mammalian cells, have enabled the large-scale production of highly immunogenic VLPs (57). These particles can present multiple epitopes in their native conformation, eliciting a strong and broad immune response (57). Advancements in adjuvant formulations and delivery systems, such as encapsulation in biodegradable nanoparticles, have also enhanced the stability and immunogenicity of VLP vaccines (58).

## 6 Brief history of antiviral development

While vaccines are essential in preventing viral infections such as FMD, antivirals are also needed to prevent the spread of infections once they occur. Therefore, there is also a growing focus on developing antiviral therapies in addition to vaccines. There have been significant advancements since the discovery of the first antiviral agents, transforming the management of viral infections. Antiviral therapy was first reported in the mid-20th century with the identification of compounds capable of inhibiting viral replication. Idoxuridine, one of the first antiviral drugs, was developed in the 1950s to treat herpes simplex virus (HSV) infections. This drug marked the beginning of targeted antiviral therapy, paving the way for the development of more specific and effective antiviral agents (59). The 1990s marked a breakthrough in human immunodeficiency virus (HIV) treatment with the introduction of highly active antiretroviral therapy (HAART). Using a combination of antiretroviral drugs that target various stages of the HIV life cycle, HAART transformed HIV from a life-threatening illness into a manageable chronic condition (60). In the late 20th century, antiviral research expanded to include broad-spectrum agents capable of targeting multiple viruses (61). Ribavirin, a nucleoside analog effective against a range of RNA viruses, has been used to treat various viral infections, including hepatitis C (62), respiratory syncytial virus (RSV) (63), and certain viral hemorrhagic fevers. The approval of ribavirin treatment highlighted the potential of broad-spectrum antivirals in managing several viral diseases (64).

As the pursuit of better and safer antiviral agents continued, the library of potential antiviral agents expanded. Now, these antiviral agents can be grouped into two broad categories. i.e., direct virus-targeting antivirals or cell-targeting antivirals have been investigated. Examples of direct virus-targeting antivirals include influenza A virus M2 ion channel inhibitors such as Amantadine, Neuraminidase inhibitors such as Oseltamivir, or hepatitis C virus non-structural protein inhibitors such as Boceprevir, Ombitasvir (65, 66). Examples of cell-targeting antiviral include Lauryl Gallate, valproic acid inhibiting FMDV, African swine fever virus and VSV (67).

Rapid advancements in molecular biology and biotechnology have fueled innovations in antiviral research. Studies are exploring the potential of RNA-based therapies, such as small interfering RNAs (siRNAs) and antisense oligonucleotides, to silence viral genes and inhibit replication. Moreover, the CRISPR–Cas9 gene-editing technology holds promise for directly targeting and eliminating viral genomes from infected cells (68).

## 7 Advancements in antivirals targeting FMDV

Considering FMDV’s high transmissibility and economic impact, developing antiviral drugs specifically targeting the virus is crucial. This section presents different classes of antiviral drugs that have shown potential FMDV-targeting activity, highlighting their mechanisms of action and efficacy. Antivirals that have been tested *in vitro, in vivo*, or both against FMDV are summarized in Table 1, and their mechanisms of action are illustrated in Figure 1.

**Table 1:** Classification of antiviral agents against foot-and-mouth disease virus.

Classification	Antiviral agent group	Mechanism of action	Examples	Ref.
Small molecule inhibitors	Nucleoside analog antiviral agents	Inhibiting the synthesis of viral RNA and proteins.	T-1105 (Pyrazine-Carboxamide Derivative)	(70)
			Ribavirin	(94)
	IMP dehydrogenase inhibitors	inhibiting inosine monophosphate dehydrogenase (IMPDH).	Merimepodib	(75)
	TLR7 (Toll-Like Receptor 7) agonists	stimulating the immune system to recognize and respond to viral infections.	Vesatolimod	(80)
	IMPDH and DHODH Inhibitors	Targeting enzymes involved in nucleotide biosynthesis.	IMPDH Inhibitors (AVN-944 and mycophenolate mofetil) and DHODH Inhibitors (teriflunomide)	(81)
	Multikinase inhibitor	inhibits tyrosine protein kinases and serine/threonine protein kinases	Sorafenib	(85)
Biologics	Interferons	Modulate immune response	Porcine IFN-*α*-Fc	(102)
Natural products	Plant alkaloids	Inhibit protein synthesis, interfere with viral replication	Homoharringtonine	(108)
	Flavonoids	Inhibit viral replication, modulate immune response	Quercetin	(109)
	Insects Venom	Induce antiviral state, enhance immune response	Bee Venom	(112)
RNA-based therapeutics	Short-hairpin RNAs (shRNAs)	Target viral RNA through RNA interference	Various shRNAs	(116)
	Artificial microRNAs (amiRNAs)	Customizable to target specific viral strains	Various amiRNAs	(116)
	Synthetic non-coding RNAs	Mimic natural RNA sequences, induction of innate immune response	FMDV ncRNAs	(121)
Gene delivery systems (viral vectors)	Baculovirus vectors (BacMam)	Introducing antiviral genes into mammalian cells	BacMam expressing glycosylated IFN-α	(101)
Pyrimidine synthesis inhibitors	Dihydroorotate Dehydrogenase (DHODH) Inhibitors	inhibiting (DHODH), involved in the *de novo* pyrimidine biosynthesis pathway	Brequinar	(125)
Immunosuppressive agents	Inosine Monophosphate Dehydrogenase (IMPDH) Inhibitors	inhibiting inosine monophosphate dehydrogenase (IMPDH) and guanosine monophosphate synthetase	Mizoribine	(127)
Antiparasitic agent	Macrocyclic Lactone	inhibiting the nuclear transport of viral proteins	Ivermectin	(98)
Minerals/Trace elements	Manganese ions (Mn^2+^)	Modulate immune response, influence viral infections	Manganese	(135)
Growth factors	Fibroblast growth factors	Regulate gene expression, inhibit viral replication	FGF11	(136)
Nanomaterials	Bimetallic nanoparticles	Disrupt viral membranes, generate reactive oxygen species	Ag-CuO nanoparticles	(139)
Virus protease inhibitors	Protease inhibitors	Inhibit viral protease 3Cpro activity	Rupintrivir	(142)

### 7.1 Small molecule inhibitors

Small molecule inhibitors include diverse chemotherapeutic agents known for their ability to affect various stages of the viral life cycle. They are broadly classified as selective small molecule kinase inhibitors, selective small molecule nonkinase inhibitors, and multikinase small molecule inhibitors (69).

#### 7.1.1 T-1105, a pyrazine-carboxamide derivative

T-1105, a derivative of pyrazine–carboxamide, is an antiviral agent that is currently being explored for its efficacy against various RNA viruses. It inhibits viral RNA polymerase, thereby preventing viral replication.

In 2022, Nishi et al. (70) demonstrated that T-1105 effectively inhibited the replication of 28 FMDV reference strains across all 7 serotypes. In domestic pigs infected with a porcinophilic FMDV serotype O (topotype CATHAY), treatment with T-1105 resulted in no clinical signs of FMD. At 48 h after inoculation, no infectious FMDV or FMDV-specific genes were detected in the sera, oral or nasal discharges, or tissues, suggesting the compound’s potential for controlling FMDV spread in pigs.

#### 7.1.2 Merimepodib

Merimepodib (MMPD, VX-497) has antiviral and immunosuppressive activities *in vitro* and *in vivo.* MMPD selectively inhibits inosine monophosphate dehydrogenase (IMPDH), thus reducing the production of guanine nucleotides essential for RNA and DNA synthesis (71). Merimepodib exhibits antiviral activity against a wide range of RNA and DNA viruses, including hepatitis C virus (HCV), hepatitis B virus (HBV), HSV type 1, human cytomegalovirus, RSV, Venezuelan equine encephalomyelitis virus (72) Zika virus (73) and SARS-CoV-2 virus (74).

The antiviral activity of MMPD against the O and A serotypes of FMDV was demonstrated *in vitro* and *in vivo* (75). MMPD provided dose-dependent inhibition of the FMDV O and A serotypes, with IC50 values of 7.859 μM and 2.876 μM, respectively. MMPD also significantly prolonged survival in FMDV-infected suckling mice, emphasizing its potential as a novel antiviral agent against FMDV.

#### 7.1.3 Vesatolimod

Vesatolimod (GS-9620) is a Toll-like receptor 7 (TLR-7) agonist that stimulates antiviral immune responses by activating TLR-7, an endosomal innate immune sensor. This activation triggers the production of type I interferon (IFN) and other inflammatory cytokines, enhancing the host’s antiviral defense mechanisms (76). The drug is being used in clinical therapy for chronic hepatitis B and HIV (77, 78). Studies have also demonstrated its antiviral efficacy against human hepatitis C virus and enterovirus 71 (77, 79). Given its broad antiviral activity, its antiviral potential against FMDV serotype O was investigated *in vitro* and *in vivo* (80). *In vitro*, vesatolimod caused more than a 100-fold reduction in FMDV load compared to ribavirin. In mice, vesatolimod conferred protection against FMDV for up to 5 days postinjection, accompanied with detectable levels of cytokines such as IL-6, IL-12, IFN-*γ*, and IFN-γ inducible protein-10. Combining vesatolimod with an inactivated FMD vaccine proved highly effective for early protection in mice.

#### 7.1.4 IMPDH and DHODH inhibitors

Inosine monophosphate dehydrogenase (IMPDH) and dihydroorotate dehydrogenase (DHODH) are essential enzymes that play crucial roles in the *de novo* synthesis of purines and pyrimidines, respectively. Inhibitors of IMPDH and DHODH ultimately lead to depletion of guanine and uridine nucleotides of cells thereby inhibiting RNA and DNA synthesis. AVN-944 and mycophenolate mofetil target IMPDH, while teriflunomide targets DHODH. These enzymes are involved in nucleotide biosynthesis, and their inhibition disrupts viral replication. Mei-Jiao et al. (81) reported that IMPDH inhibitors (AVN-944 and mycophenolate mofetil) and the DHODH inhibitor (teriflunomide) effectively suppressed FMDV serotypes O and A in IBRS-2 cells in a dose- and serotype-dependent manner. The antiviral effects were most pronounced during the early stages of infection (0–8 h post-infection). Furthermore, treatment with AVN-944 and teriflunomide significantly increased the survival rates of FMDV-infected mice, demonstrating their broad-spectrum antiviral potential. However, concerns about toxicity and potential side effects due to interference with host cellular processes remain. Additionally, serotype-specific responses may limit their universal application. Future research should focus on optimizing dosages, assessing long-term safety, developing serotype-targeted therapies, and exploring combination treatments to enhance their antiviral efficacy.

#### 7.1.5 Sorafenib

Sorafenib, a bi-aryl-urea compound, is a multiprotein kinase inhibitor that is well known for its anticancer and antiviral activities (82). It inhibits tumor cell proliferation by blocking Raf-1, B-Raf, and kinase activities in the Ras/Raf/MEK/ERK signaling pathways while also reducing tumor angiogenesis by targeting vascular endothelial growth factor receptors, platelet-derived growth factor receptors, and hepatocyte factor receptor (c-KIT) (83, 84). Theerawatanasirikul et al. explored the antiviral activity of sorafenib against FMDV (85). They reported that sorafenib effectively reduced FMDV replication in a dose-dependent manner, with EC_50_ values of 2.46 μM at the previral entry stage and 2.03 μM at the postviral entry stage. Molecular docking analysis indicated that sorafenib binds to the critical catalytic residues (D245, D338, S298, and N307) in the active site of FMDV 3DPol. Furthermore, sorafenib inhibited the c-RAF, AKT, and PI3K pathways, which are vital for the viral life cycle. The main concern with sorafenib is its potential toxicity, as it also affects host cell signaling pathways involved in cell proliferation and survival. Further clinical trials are needed to confirm its real-world effectiveness in treating FMDV.

#### 7.1.6 Ribavirin

Ribavirin (1-ribosyltriazole), a guanosine analog, is well known for its broad-spectrum antiviral activity against many RNA and DNA viruses (86). Multiple mechanisms have been proposed to explain the antiviral activity of ribavirin. The drug inhibits RNA-dependent RNA polymerase and IMPDH enzyme activities and upregulates IFN-stimulated genes (ISGs) (87). Ribavirin has been used to treat infections caused by respiratory syncytial virus (RSV) in children, as well as Lassa fever virus, influenza A and B, and hepatitis C virus infections (88–91). Several studies have demonstrated its potent antiviral efficacy against various FMDV serotypes *in vivo* and *in vitro*. Soumajit et al. (92) showed that ribavirin inhibited the *in vitro* replication of FMDV serotypes O, A and Asia 1. Choi et al. (93) demonstrated that ribavirin effectively inhibited FMDV *in vitro* and *in vivo. In vivo,* studies in mice and pigs demonstrated that oral antiviral treatment complemented with a vaccine led to synergistically enhanced antiviral activity against FMDV. Nikunjkumar et al. (94) systematically investigated ribavirin’s antiviral efficacy against FMDV in suckling and adult C57BL/6 mice and revealed that ribavirin significantly reduced viral titers and conferred protection against FMDV infection.

#### 7.1.7 Small molecule inhibitors targeting FMDV 3Cpro

FMDV 3Cpro plays a crucial role in viral replication by cleaving the viral polyprotein into mature, functional proteins. Given its essential function and high degree of conservation across FMDV serotypes, 3Cpro is considered an ideal target for antiviral drug development. Small molecule inhibitors targeting FMDV 3Cpro disrupt the viral life cycle and inhibit replication. Kim et al. (95) screened an in-house library of small molecule inhibitors targeting FMDV 3Cpro and identified potent inhibitors based on aldehyde and *α*-ketoamide structures. Their structure–activity relationship studies demonstrated that these compounds are highly effective in enzyme- and cell-based assays. Theerawatanasirikul S. et al. (96) used molecular docking studies to screen potential 3Cpro inhibitors. Of the seven selected compounds, NSC116640 and NSC332670 showed strong *in vitro* inhibition of FMDV replication.

These studies highlight the potential of various small-molecule antiviral agents in controlling FMDV, providing a foundation for developing effective therapeutics against this economically significant virus.

### 7.2 Biologics

Biologics, including monoclonal antibodies, interferons (IFNs), and vaccines, are emerging as effective antivirals against FMDV. These biologics act by either directly neutralizing the virus or enhancing the host’s immune response.

#### 7.2.1 Porcine INF-*α* linked to porcine IgG-Fc

Type I IFNs are produced rapidly following viral infection, eliciting a host innate immune response involving the stimulation/production of several IFN-stimulated genes, some of which possess antiviral activity. IFNs have been successfully used to treat viral infections such as RSV and hepatitis B and C infections (97). A previous study showed that pretreatment with IFN-*α*/*β* significantly inhibited FMDV replication in cell culture (98). However, the efficacy of this approach is limited by the short half-life of IFN in the host. To overcome this limitation, Chinsagaram et al. developed a replication-defective human adenovirus type 5 vector containing porcine IFN-*α* genes (Ad5-pIFNα). They demonstrated that at 24 h post-challenge, treatment with Ad5-pIFN*α* conferred complete protection from clinical disease in pigs (99). Other approaches have been developed to boost the antiviral effects of IFN against FMDV. Pegylated porcine IFN-*α* protein (100) and highly glycosylated porcine IFN-*α* protein (101) have proven extremely effective in conferring protection against FMDV challenge in pigs. Recently, Kim et al. (102) reported a further advancement in the use of porcine IFN-α–based therapy. They demonstrated the broad antiviral activity of the porcine IFN-α–IgG-Fc fusion protein against seven serotypes of FMDV, *in vitro* and in pig models. They also showed that mice receiving the combination of porcine IFN-α-Fc and an inactivated vaccine showed increased neutralizing titers compared with those receiving the vaccine alone.

### 7.3 Natural products

Natural products have gained attention as potential antiviral agents due to their diverse bioactive properties and lower likelihood of inducing resistance. Various natural compounds, including essential oils, plant extracts, and bee venom, have demonstrated promising efficacy against FMDV. Compounds such as flavonoids, alkaloids, and terpenoids can inhibit FMDV replication by targeting viral entry, replication, or assembly.

#### 7.3.1 Homoharringtonine

Homoharringtonine, a plant alkaloid and protein synthesis inhibitor, blocks translation elongation by binding to the ribosome and preventing the correct positioning of aminoacyl-tRNA, thereby interfering with viral replication. Homoharringtonine has demonstrated broad antiviral activity against various viruses such as HBV, bovine viral diarrhea virus (BVDV), chikungunya virus, varicella–zoster virus, vesicular stomatitis virus (VSV), Newcastle disease virus (NDV), porcine epidemic diarrhea virus, and HSV-1 (103–107). Due to the broad antiviral activity of Homoharringtonine, Gong et al. (108) tested the antiviral activity of homoharringtonine against serotypes O and A *in vitro* and revealed strong inhibition of FMDV strains (O/MYA98/BY/2010 and A/GD/MM/2013) in swine kidney cells (IBRS-2) during the early stages of infection. They further proposed *in vivo* testing of the drug. The main advantage of homoharringtonine is its broad-spectrum antiviral potential. However, its use is limited by potential toxicity and adverse effects on host cell protein synthesis. Future research should focus on optimizing dosages, evaluating long-term safety, and conducting *in vivo* studies to assess its efficacy in field settings.

#### 7.3.2 Quercetin

Quercetin is a flavonoid with broad-spectrum antiviral activity at various stages of viral replication and immune-modulating activity. A study by Lee et al. (109) revealed that quercetin induced dose-dependent inhibition of FMDV (serotype O) in porcine kidney (LFBK) cells while also upregulating IFN-*α* and ISGs. When combined with an inactivated FMD vaccine, quercetin increased the production of several cytokines, enhanced survival rates, and neutralized antibody titers in mice, thus acting as an adjuvant. The main advantage of quercetin is its dual action in both antiviral activity and immune enhancement. However, its effectiveness may vary depending on the viral strain. Future research should focus on testing its antiviral effect on more FMDV strains.

#### 7.3.3 Bee venom

The antiviral properties of bee venom are attributed to its complex mixture of peptides and enzymes, including melittin, apamin, and phospholipase A2. These components have demonstrated efficacy against mainly enveloped viruses through mechanisms such as disrupting viral membranes, inhibiting viral replication, or modulating the immune response (110, 111).

The activity of bee venom against FMDV seems to be multipronged. Direct treatment of FMDV with bee venom resulted in a 25.7% reduction in viral titers, demonstrating its virucidal activity. Additionally, bee venom significantly inhibited viral replication in treated cells. A notable increase in IFN-*γ* levels suggested that bee venom induced an antiviral state. (112). Another study showed that bee venom induced long-lasting, specific immunity against FMDV if inoculated with the FMD vaccine (113).

### 7.4 RNA-based strategies

The first report of using antisense oligonucleotide against Rous sarcoma virus paved the way for innovative RNA-based therapeutic strategies to combat a range of viral infections (114). These include using RNA molecules that directly interfere with virus replication or modulating the host immune response.

#### 7.4.1 RNAi-based strategies

RNAi regulates gene expression by silencing sequence-specific posttranscriptional genes. It also serves as a cellular defense mechanism against viral infections. RNAi has shown promising *in vitro* results against IAV, HBV, Ebolavirus, HIV, and RSV. Interestingly, RNAi-based therapies against RSV, HBV and HIV are in different phases of clinical trials (115). Therefore, RNA interference-based approaches to control viral infections have also opened up a new avenue for controlling FMD.

Currá et al. (116) identified three potential target sequences within the FMDV 3D coding region and evaluated the antiviral potential of short-hairpin RNAs (shRNAs) and artificial microRNAs (amiRNAs). They reported that shRNAs and amiRNAs caused 70–95% inhibition of FMDV in BHK-21 cells. Santos et al. demonstrated that using a shRNA targeting the 2B nonstructural protein coding region of FMDV RNA in porcine cells significantly reduced viral titers and protein levels. This shRNA approach was effective against multiple FMDV serotypes (117). Recently, Sahu et al. (118) conducted a comprehensive database search of microRNAs (miRNAs) and identified 12 mature host miRNAs that targeted 284 sites across 98 distinct FMDV genomic sequences. Among these, eight miRNAs significantly reduced virus titers in BHK-21 cells.

#### 7.4.2 Non-coding RNA-based strategies

Noncoding synthetic RNAs (ncRNA) are RNA-based antiviral agents designed to mimic natural RNA sequences. They interfere with viral replication response by targeting specific viral RNA sequences (114) or modulate the host immune system. ncRNAs that mimic in sequence and structure of three different domains in the non-coding regions of FMDV genome have been shown to induce antiviral immune responses via Toll-like receptor and retinoic acid-inducible gene-I (RIG-I) pathways (119, 120). Rodríguez-Pulido et al. (121) showed that these ncRNAs induced strong type I IFN-dependent antiviral activity in porcine cultured cells and mice, independent of FMFDV serotypes. These results demonstrate the biological activity of ncRNAs in FMDV host cells and their potential for conferring protection against FMDV.

### 7.5 Gene delivery systems (viral vector)

Gene delivery systems using viral vectors have demonstrated significant potential as FMD antiviral approaches. Adenoviruses, lentiviruses, and adeno-associated viruses are modified to carry genetic material into host cells, enabling the expression of antiviral genes or RNA molecules that can disrupt viral replication and enhance the host’s immune response to the virus. These vectors can carry genes encoding antiviral proteins, siRNAs, or other therapeutic RNAs targeting FMDV.

#### 7.5.1 BacMam expressing highly glycosylated porcine IFN-*α*

BacMam is a baculovirus *Autographa californica* nuclear polyhedrosis virus (AcMNPV)-based viral vector used as a mammalian cell gene delivery vector. Kord et al. (122), first reported the successful use of a BacMam-based vaccine against HCV infection, showing that the vaccine triggered cellular and humoral immunity against HCV. The antibodies against the HCV surface E2 glycoprotein displayed strong cross-neutralizing activities. Kim et al. (101) developed a recombinant BacMam vector expressing consensus porcine IFN-*α* with three additional N-glycosylation sites (Bac-Con3N IFN-α). This vector facilitated enhanced protein expression in mammalian cells and induced high levels of IFN-α *in vitro* and *in vivo*. Moreover, Bac-Con3N IFN-α demonstrated *in vivo* antiviral activity and adjuvant effects when combined with an inactivated FMD vaccine in pigs. Thus, Bac-Con3N IFN-α holds promise as an antiviral and adjuvant agent, enhancing protection against FMDV when used alongside inactivated FMD vaccines in pigs.

### 7.6 Immunosuppressive agents

#### 7.6.1 Brequinar

Brequinar, known for its antiviral and anticancer properties, is primarily a potent selective inhibitor of dihydroorotate dehydrogenase (DHODH), an enzyme critical for *de novo* pyrimidine synthesis. Brequinar is a host-acting antiviral compound with demonstrated antiviral activity against HIV-1 (123), COVID-19 (124) dengue virus, VSV, ZIKA virus, hepatitis E virus, and FMDV.

Li et al. (125) demonstrated the *in vitro* antiviral activity of brequinar in swine kidney cells (IBRS-2) against the FMDV O and A serotypes. This effect was validated in a mouse model, where a 50 μg dose of brequinar conferred 25% protection for 5 days post-FMDV exposure, indicating its potential as an effective antiviral agent against FMD. However, its effectiveness against FMDV is limited, as seen by the modest protection observed in animal models. Additionally, concerns about potential toxicity and long-term safety arise from its impact on host cell metabolism. Future research should focus on improving its efficacy against FMDV through optimized dosing and evaluating its safety profile.

#### 7.6.2 Mizoribine

Mizoribine, a nucleoside analog, is an immunosuppressive agent that inhibits lymphocyte proliferation by targeting inosine monophosphate dehydrogenase and guanosine monophosphate synthetase. It has shown synergistic anti-CMV activity when combined with antiviral agents like ganciclovir and ribavirin (126).

Mizoribine effectively inhibited FMDV replication in IBRS-2 cells (127). A time-of-drug-addition assay showed that mizoribine targets an early phase of the viral replication cycle. Antiviral efficacy against FMDV was also demonstrated *in vivo*, highlighting its potential as a therapeutic agent for FMDV infections. However, its immunosuppressive properties could limit its use, potentially increasing susceptibility to secondary infections. Future research should focus on optimizing its therapeutic potential and assessing its safety.

### 7.7 Macrocyclic lactones

The subfamilies of macrocyclic lactones are avermectin and milbemycin. Avermectins include ivermectin, abamectin, doramectin, and selamectin; the milbemycin subfamily includes moxidectin, milbemycin oxime, and nemadectin. Ivermectin, moxidectin, and milbemycin oxime are used in veterinary medicine (128, 129).

#### 7.7.1 Ivermectin

Ivermectin is a popular antiparasitic drug used worldwide, making it one of the most important global health medicines. Its creators were awarded the Nobel Prize in medicine in 2015. Since its first approval for animal use in 1981, the drug has demonstrated efficacy *in vitro* and *in vivo* against nematodes, arthropods, mycobacteria, and viruses such as SARS-CoV-2, Zika virus, pseudorabies virus, and porcine circovirus 2 (130, 131).

Naeem et al. (132) investigated the antiviral potential of ivermectin against FMDV serotypes O, A, and Asia-1. Ivermectin decreased viral titers by two to three logs in BHK-21 cells at 2.5 μM and 5 μM. The greatest reduction in viral titer occurred during the replication phase, with less impact observed during attachment and entry phases. This research highlights the potential of ivermectin as an effective *in vitro* treatment for FMDV and suggests that it could be valuable in managing FMDV infections.

### 7.8 Minerals/trace elements

#### 7.8.1 Manganese (Mn)

Mn is a crucial enzyme cofactor that plays roles in different physiological processes, such as synthesis and metabolism, immunity, development, reproduction, and neuronal function, and acts as an antioxidant (133). It exerts its antiviral effects by modulating the innate immune system. A previous study showed that Mn exerted its antiviral effects against DNA viruses via the cGAS–STING pathway (134). However, Zhang et al. (135) reported that the effect of Mn on RNA viruses, specifically FMDV, was mediated by activating NF-κB and upregulating ISGs in a cGAS–STING pathway-independent manner. Animal experiments showed that Mn^2+^ effectively conferred protection to C57BL/6 N mice from FMDV infection, suggesting its potential utility in antiviral defense.

### 7.9 Novel antiviral approaches

#### 7.9.1 Fibroblast growth factor 11 (FGF11)

In addition to its role in cell growth and differentiation, FGF11 possesses antiviral activity. Kang et al. (136) reported that FGF11 inhibits transcription and translation in FMDV. A plasmid reporter system was constructed by linking the FMDV 5′-UTR, which contains the *cis*-acting replication element and internal ribosome entry site, with a luciferase reporter gene. FGF11 was found to reduce the expression of the FMDV 5′-UTR–luciferase reporter gene in a dose-dependent manner. Additionally, FGF11 suppressed FMDV gene expression and replication in infected pig cells, reducing RNA production by FMDV RNA polymerase 3D and significantly reducing the cytopathic effects of FMD. These findings suggest that FGF11 can be used as an intervention approach against FMDV pathogenesis and transmission.

#### 7.9.2 Nanomaterial-based antiviral agents

Bimetallic silver–copper oxide nanoparticles are nanomaterial-based antiviral agents that utilize the combined properties of silver and copper to disrupt viral membranes, generate reactive oxygen species, and bind to viral particles, thus inhibiting viral replication and conferring broad-spectrum antiviral protection (137, 138). El-Batal et al. (139) synthesized bimetallic silver–copper oxide nanoparticles using gamma irradiation, with gum Arabic polymer serving as a capping and reducing agent. The nanoparticles were characterized by UV–Vis spectroscopy, high-resolution transmission electron microscopy, scanning electron microscopy, dynamic light scattering, and X-ray diffraction, and their antimicrobial and antibiofilm properties were assessed. Clinical investigations of cows and buffaloes revealed ulcerative lesions on the mouth and interdigital regions, which healed within a week of topical application of silver–copper oxide nanoparticles. Cytotoxic assays showed the nanoparticles’ protective effects on BHK-21 cells before viral infection, demonstrating the inhibition of FMDV. Future research should focus on refining nanoparticle formulations and assessing long-term safety.

#### 7.9.3 Virus protease inhibitors

Protease inhibitors act as enzyme-targeted antiviral agents by targeting viral proteases, which play a vital role in the viral life cycle by cleaving polyproteins into functional components (140). Specifically, FMDV utilizes Lpro and 3Cpro proteases to degrade cGAS in swine cells, thereby disrupting the cGAS–STING-mediated antiviral response (141). This degradation impairs innate immunity, allowing the virus to evade host defenses. Notably, inhibiting the cGAS–STING pathway was found to enhance viral replication, underscoring the pathway’s critical role in antiviral defense. Given this, protease inhibitors like rupintrivir, a well-characterized 3Cpro inhibitor, was shown to prevent cGAS degradation, restore innate immune signaling, and suppress FMDV replication (142). Developing specific 3Cpro inhibitors and combining them with STING agonists presents a potential therapeutic approach to counteract FMDV immune evasion and strengthen antiviral defenses.

## 8 Challenges in developing antivirals against FMDV

Animal models are essential for developing antivirals against FMDV as they provide critical insights into the pharmacokinetics, efficacy, and safety profiles of potential drugs. The efficacy of antiviral agents is assessed through challenge studies, which measure the reduction in viral load and the alleviation of clinical signs of FMD. Additionally, *in vivo* studies play a key role in determining the safety of these drugs by monitoring animals for adverse effects and toxicities, ensuring their suitability for use in livestock. Animal models such as pigs and cattle are particularly valuable as they bridge the gap between *in vitro* findings and practical applications, offering a comprehensive understanding of antiviral performance.

However, several challenges persist in developing effective antivirals against FMDV. One major obstacle is the emergence of drug-resistant viral strains due to the virus’s continuous evolution, thus complicating treatment and necessitating ongoing adaptation of antiviral approaches. One way to reduce the rate of emergence of resistant viral strains is to use cell-targeting antivirals (143).

Another occasionally faced challenge is that many antiviral compounds that show promise *in vitro* fail to achieve similar success *in vivo* due to factors such as pharmacokinetics, drug delivery challenges, and host immune responses. Furthermore, the lack of approved antiviral drugs for FMD in livestock highlights a significant gap in current research, mainly due to the high production costs of antiviral drugs for animal use, as the cost/effect ratio in veterinary medicine is less favorable compared to human treatments. This limits the adoption of antiviral agents, particularly in developing countries. To address these challenges, it is crucial to reduce production costs while maintaining efficacy, ensuring that antiviral therapies are economically viable for large-scale use in the veterinary industry.

The potential combination of antiviral compounds with vaccines against FMD offers a promising strategy to minimize the susceptibility window and achieve early effective protection. By combining antivirals with vaccines, it is possible to enhance both immediate and long-term protection against FMDV infections. Innate immunity activators represent a critical aspect of this approach. Borrego et al. reported that pigs inoculated with FMD vaccines and ncRNAs developed better T-cell responses than those inoculated with vaccines alone (144). Mice receiving FMD vaccine with ncrRNAs developed earlier, higher and longer B and T cell responses compared to the mice receiving FMD vaccine only. This study demonstrates that strategies involving activating innate immune responses along with adaptive immune responses can generate a more robust, specific and lasting immune response.

## 9 Future directions in antiviral approaches against FMDV

Advancing antivirals against FMDV involves several promising approaches. Combination therapies, for instance, can improve treatment effectiveness and minimize the risk of resistance development by targeting multiple stages of the viral life cycle or using drugs with different mechanisms of action. Drug repurposing is another valuable approach, where existing medications with established safety profiles are adapted for new antiviral purposes. For example, ivermectin, traditionally used as an antiparasitic, has demonstrated potential antiviral activity against FMDV. Additionally, novel drug discovery methods, driven by advancements in biotechnology and molecular modeling, are opening new avenues for developing antiviral agents. Techniques like high-throughput screening, structure-based drug design, and RNA interference (RNAi) enable the identification of innovative targets and therapeutic compounds to combat FMDV more effectively.

## 10 Conclusion

Antiviral drugs have significant potential as a supplementary approach for managing FMD outbreaks and reducing their devastating socioeconomic consequences. Current antiviral candidates’ diverse mechanisms of action - from inhibiting viral attachment and entry to suppressing replication and gene expression - offer targeted approaches to disrupt the critical stages of the FMDV lifecycle. These interventions enhance existing vaccination programs and provide immediate control options in situations where vaccines may be unavailable, ineffective against emerging strains, or delayed in deployment.

Ongoing research is essential to refine these antiviral agents, addressing challenges such as viral resistance, host toxicity, and large-scale production. Efforts must focus on optimizing drug efficacy through structure-based drug design, exploring combination therapies to enhance antiviral potency and reduce resistance, and developing innovative delivery systems to ensure practical application in the field. Moreover, identifying and characterizing new therapeutic targets, such as viral proteases and host factors critical for viral replication, could expand the arsenal of effective treatments.

Enhanced global readiness and response to FMD outbreaks also require the integration of antiviral solutions into comprehensive disease management plans. This includes developing robust surveillance systems to detect outbreaks early, evaluating antiviral drugs under field conditions, and ensuring accessibility for resource-limited regions that substantially rely on livestock industries. By addressing these challenges, the scientific community can develop effective, sustainable strategies to control FMD, safeguard global food security, and protect the livelihoods of millions who are dependent on livestock production.

## Glossary

FMD: Foot-and-mouth disease
FMDV: Foot-and-mouth disease virus
RNA: Ribonucleic acid
DNA: Deoxyribonucleic acid
VLPs: virus-like particles
HSV: herpes simplex virus
RSV: respiratory syncytial virus
HIV: human immunodeficiency virus
HAART: highly active antiretroviral therapy
IAV: influenza A virus
siRNAs: small interfering RNAs
MMPD: Merimepodib
IMPDH: inosine monophosphate dehydrogenase
HCV: hepatitis C virus
HBV: hepatitis B virus
SARS-CoV-2: Severe acute respiratory syndrome coronavirus 2
TLR-7: Toll-like receptor 7
IFN: interferon
DHODH: dihydroorotate dehydrogenase
ISGs: IFN-stimulated genes
Ad5-pIFN*α*: adenovirus type 5 vector containing porcine IFN-α genes
BVDV: bovine viral diarrhea virus
VSV: vesicular stomatitis virus
NDV: Newcastle disease virus
RNAi: RNA interference
shRNAs: short-hairpin RNAs
amiRNAs: artificial microRNAs
ncRNAs: noncoding RNAs
CMV: Cytomegalovirus
FGF11: Fibroblast growth factor 11
