# Correction: Advancements in antiviral approaches against foot-and-mouth disease virus: a comprehensive review

**DOI:** 10.3389/fvets.2025.1683089

**Published:** 2025-10-01

**Authors:** Mahmoud Mohamadin, Rashid Manzoor, Ahmed Elolimy, Mohamed Abdelmegeid, Samah Mosad, Sahar Abd El Rahman

**Affiliations:** ^1^Department of Virology, Faculty of Veterinary Medicine, Mansoura University, Mansoura, Egypt; ^2^College of Veterinary Medicine, University of Al Dhaid, Sharjah, United Arab Emirates; ^3^Veterinary Science Program, Faculty of Health Sciences, Higher Colleges of Technology, Sharjah, United Arab Emirates; ^4^Department of Integrative Agriculture, College of Agriculture and Veterinary Medicine, United Arab Emirates University, Al Ain, United Arab Emirates; ^5^Department of Animal Medicine, College of Veterinary Medicine, Kafrelsheikh University, Kafr El Sheikh, Egypt

**Keywords:** foot-and-mouth disease, antiviral approaches, vaccination advancements, novel therapeutics, future challenges

In the published article, there was a mistake in the affiliation of one of the co-authors, Dr. Rashid Manzoor. Dr. Rashid Manzoor's affiliation was displayed as “^2^College of Veterinary Medicine, University of Al Dhaid, Sharjah, United Arab Emirates”. The correct affiliation is “^3^Veterinary Science Program, Faculty of Health Sciences, Higher Colleges of Technology, Sharjah, United Arab Emirates.”

The original version of this article has been updated.

